# A Rare Case of Marchiafava-Bignami Disease With Reversible Splenial Lesion

**DOI:** 10.7759/cureus.81845

**Published:** 2025-04-07

**Authors:** Baishyak Renuji, Jaisurya Jaisukhalal, Jeyaseelan Nadarajah, Krishnan Balagopal

**Affiliations:** 1 Neurology, Malankara Orthodox Syrian Church (MOSC) Medical College Hospital, Ernakulam, IND; 2 General Internal Medicine, Derriford Hospital, University Hospitals Plymouth NHS Trust, Plymouth, GBR; 3 Neuroradiology, Malankara Orthodox Syrian Church (MOSC) Medical College Hospital, Ernakulam, IND

**Keywords:** alcohol related complications, corpus callosum lesion, marchiafava-bignami disease (mbd), splenium of the corpus callosum, thiamine or vitamin b1 deficiency

## Abstract

Individuals with a history of chronic alcohol consumption can develop Marchiafava-Bignami disease (MBD), a rare neurological disorder that is thought to occur secondary to thiamine deficiency and alcohol-induced brain damage. It is characterized by the toxic demyelination and necrosis of the corpus callosum.

We report the case of a 42-year-old man who developed acute-onset altered sensorium and irrelevant speech output associated with ataxia. The clinical presentation raised a suspicion of MBD, although it was not a top differential diagnosis initially. Magnetic resonance imaging revealed significant demyelination of the splenium of the corpus callosum, confirming the suspicion and prompting immediate intervention aimed at addressing both the neurological manifestations and the possible underlying thiamine deficiency. Relevant history of significant alcohol consumption further supported this diagnosis. Following the initiation of thiamine supplementation and supportive care, the patient exhibited gradual improvement in neurological function, eventually leading to a complete clinical recovery and resolution of radiological findings, suggestive of the type B spectrum of the disease.

This report highlights the importance of clinical evaluation and neuroimaging in the diagnosis, treatment, and prognostic stratification of patients with MBD.

## Introduction

Excess alcohol consumption and alcohol withdrawal are associated with a variety of neuropsychiatric syndromes, most of which are associated with significant morbidity and mortality [1]. The spectrum of alcohol-related neuropsychiatric disorders includes a wide range of conditions such as alcohol withdrawal, Wernicke’s encephalopathy, Wernicke-Korsakoff psychosis, Marchiafava-Bignami disease (MBD), alcohol-related dementia, and alcohol-related mood disorders.

MBD is a rare disorder characterized by the demyelination and tissue necrosis of the corpus callosum. This disease is commonly observed in individuals who have chronic alcohol consumption and malnutrition [2]. The exact etiology of MBD is not very clearly understood, but from the available research data, it is thought to be caused by a combination of thiamine deficiency associated with alcohol-induced neurotoxicity [3]. The incidence of MBD shows no predilection towards a particular race or ethnicity, but its prevalence is higher in males, which could be attributed to the higher alcohol consumption in males than females [2]. The symptomatology of the MBD is non-specific but typically includes impairment in consciousness associated with other focal neurological deficits. Magnetic resonance imaging of the brain is the gold standard diagnostic investigation, which characteristically shows demyelination involving the corpus callosum. Extensive cerebral cortex involvement and severe impairment in consciousness are predictors of poor outcome. In spite of a poor prognosis, some patients with MBD have been reported to show a favourable response to the administration of thiamine in comparison to high-dose corticosteroids, which have limited evidence of efficacy in MBD [2].

Our intention with this case report is to highlight the importance of clinical evaluation and neuroimaging in the diagnosis, treatment and prognostic stratification of patients with MBD, which could facilitate proper communication to the patient/relatives.

## Case presentation

A 42-year-old man was brought to the emergency department with a history of altered sensorium, irrelevant speech output, and swaying while walking for two days. He used to consume 500-750 ml of alcohol per day for the past 10 years. The last time he consumed alcohol was two days prior to the day at the hospital. There was no history of fever, headache, vomiting, or seizures. His past medical history was unremarkable for diabetes, hypertension, epilepsy, or other significant medical conditions. Clinical examination revealed a confused patient with a Glasgow Coma Scale (GCS) score of 12 (E3V3M6); the pupillary and corneal reflexes were normal with no abnormalities in extraocular movements; power and tone were normal on all limbs with no asymmetry, and sensory examination was grossly normal. Gait assessment revealed a wide-based gait with significant ataxia. There were no signs of sympathetic apraxia, ruling out an interhemispheric disconnection syndrome (assessed after neuroimaging).

**Figure 1:**
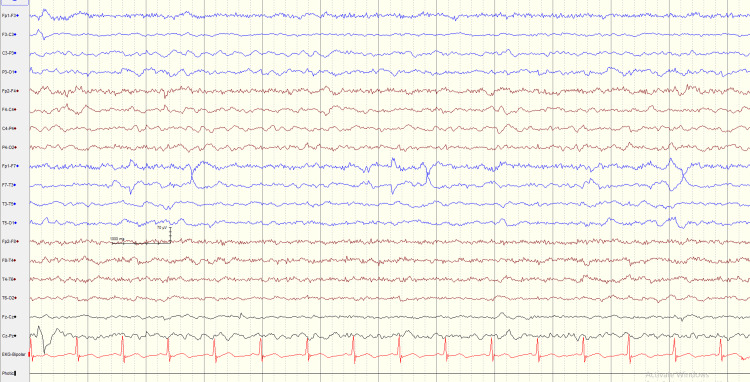
EEG The EEG capture shows intermittent slowing in the theta range.

Initially, the possibility of Wernicke’s encephalopathy and alcohol withdrawal syndrome with unwitnessed seizures (postictal phase) was suspected, and subsequently the patient was admitted for further evaluation. A CT scan of the brain was performed, which was unremarkable. A full blood count, inflammatory markers, electrolytes (sodium, potassium, calcium, and magnesium), serum ammonia, and liver function tests were done, all of which were within normal range (Table 1). A cerebrospinal fluid (CSF) analysis (including an infectious serology panel) performed did not reveal anything significant (Table 2). An EEG was also performed, which showed intermittent slowing in the theta range (Figure 1), which further helped with distinguishing it from different types of metabolic encephalopathies that prominently show generalized slowing and triphasic waves in the EEG.

**Table 1:** Blood investigations All blood investigations were normal. WBC: white blood cell; CRP: C-reactive protein; AST: aspartate transferase; ALT: alanine transaminase.

Parameters	Obtained value	Reference range
Blood haemoglobin	15	14-17 g/dL
WBC count	9	4.5-11 x 10^3^ /μL
Haematocrit	43	42-50 %
Platelets	301	150-450 x 10^3^ /μL
CRP	2	<3.0 mg/L
Serum sodium	139	135-145 mEq/L
Serum potassium	4.2	3.5-5 mEq/L
Serum calcium (corrected)	8.7	8.5-10.5 mg/dL
Serum magnesium	1.9	1.7-2.2 mg/dL
Serum urea	17	5-20 mg/dL
Serum creatinine	0.9	0.6-1.2 mg/dL
Serum glucose	72	65-100 mg/dL
Serum bilirubin	0.4	0.2-1.2 mg/dL
Serum ammonia	35	15-60 mcg/dL
AST	25	8-48 U/L
ALT	32	7-55 U/L

**Table 2:** CSF analysis Obtained values are within normal limits and CSF is negative for viral screen. CSF: cerebrospinal fluid

Characteristics	Obtained Values (Reference range)
Pressure	11 cmH_2_O (7-20 cmH_2_O)
Colour	Clear
Turbidity	Absent
Blood	Absent
Glucose	70 mg/dL (50-80 mg/dL)
Protein	31 mg/dL (15-45 mg/dL)
White blood cell counts	4 cell/mm^3^ (<5 cells/mm^3^)
CSF viral panel	Negative

**Figure 2:**
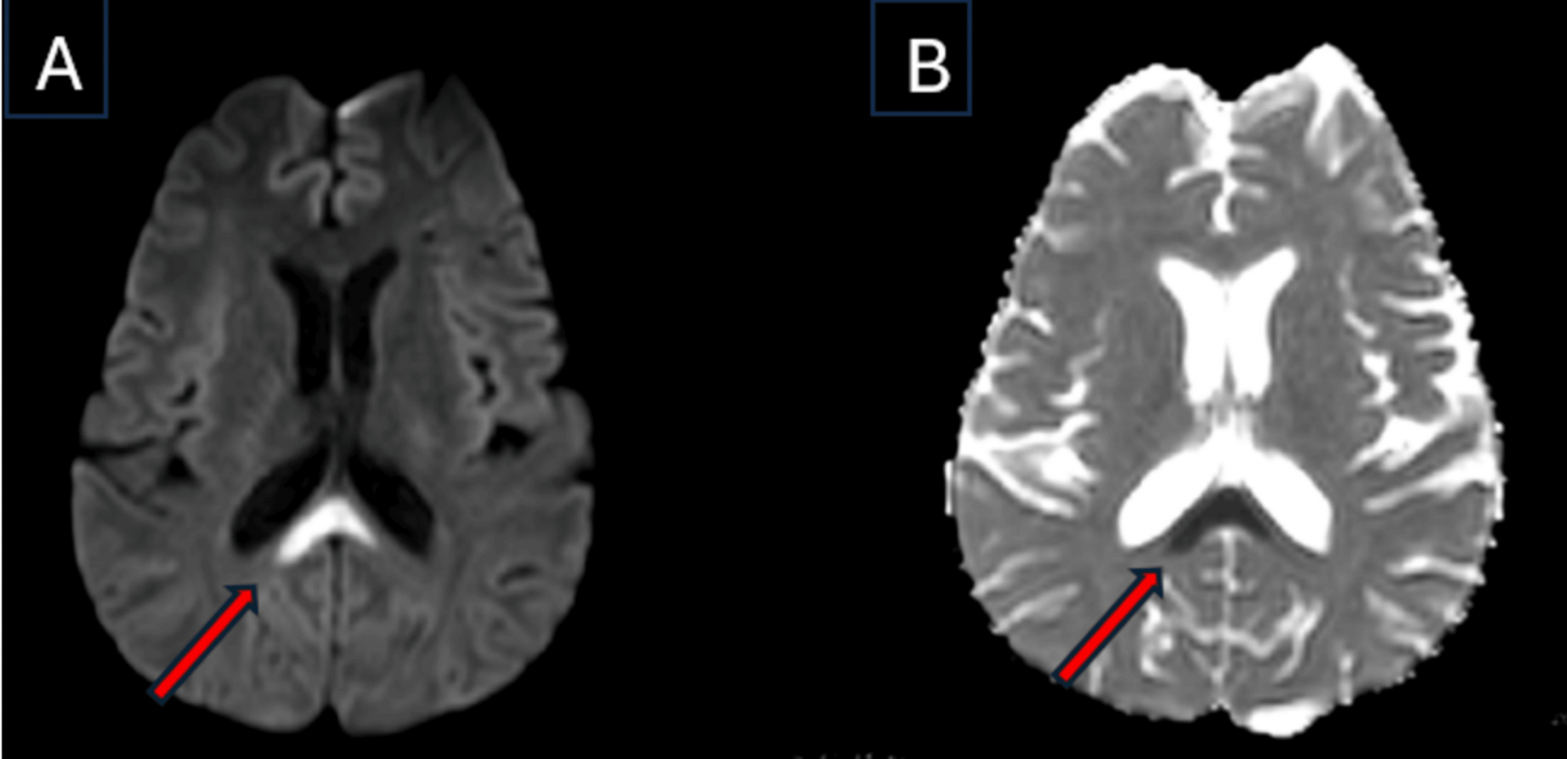
MRI Brain A) The diffusion-weighted imaging (DWI) sequence shows restricted diffusion at the splenium of the corpus callosum (red arrow), possibly suggestive of transient intra-myelinic edema in this case. This pattern is consistent with partial corpus callosum involvement typical of Marchiafava-Bignami disease (MBD) type B. B) The corresponding apparent diffusion coefficient (ADC) map shows restricted diffusion at the splenium of corpus callosum (red arrow).

**Figure 3:**
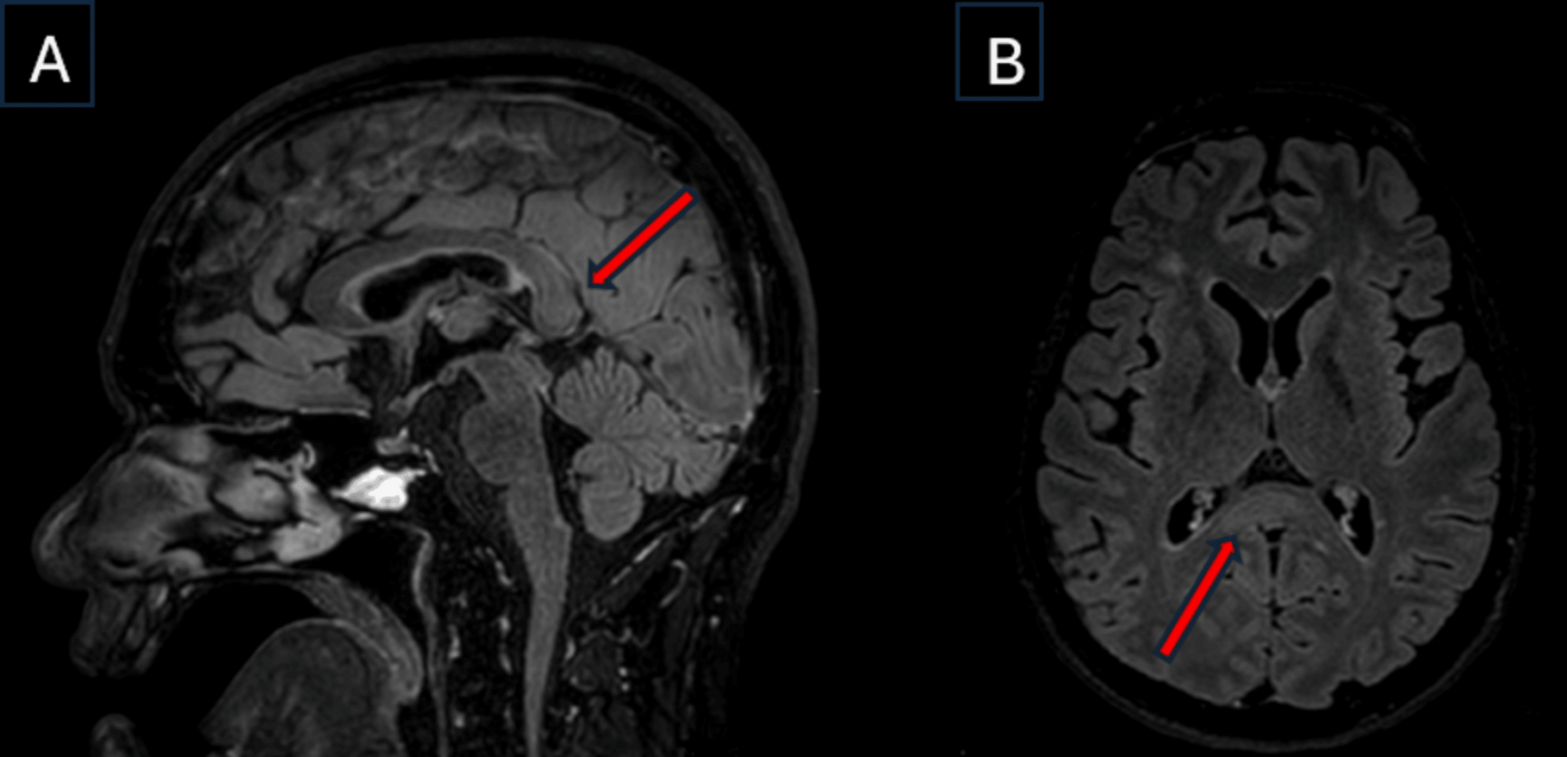
MRI Brain A) The Fluid-Attenuated Inversion Recovery (FLAIR) sequence—sagittal view—shows a hyperintense lesion at the center of the splenium of the corpus callosum, sparing the peripheral fibers—"sandwich sign" (red arrow)—signifying possible transient intra-myelinic edema. B) The FLAIR sequence—axial view—shows a hyperintense lesion at the splenium of the corpus callosum (red arrow).

Subsequently, an MRI scan of the brain with contrast was done, which showed restricted diffusion and hyperintensity in the T2/Fluid-Attenuated Inversion Recovery (FLAIR) sequences, involving the central fibers of the splenium of the corpus callosum (Figures 2, 3), which was non-enhancing with the contrast. In addition, the scan showed no evidence suggestive of Wernicke’s encephalopathy, such as symmetrical involvement of the thalami or the mammillary bodies.

**Figure 4:**
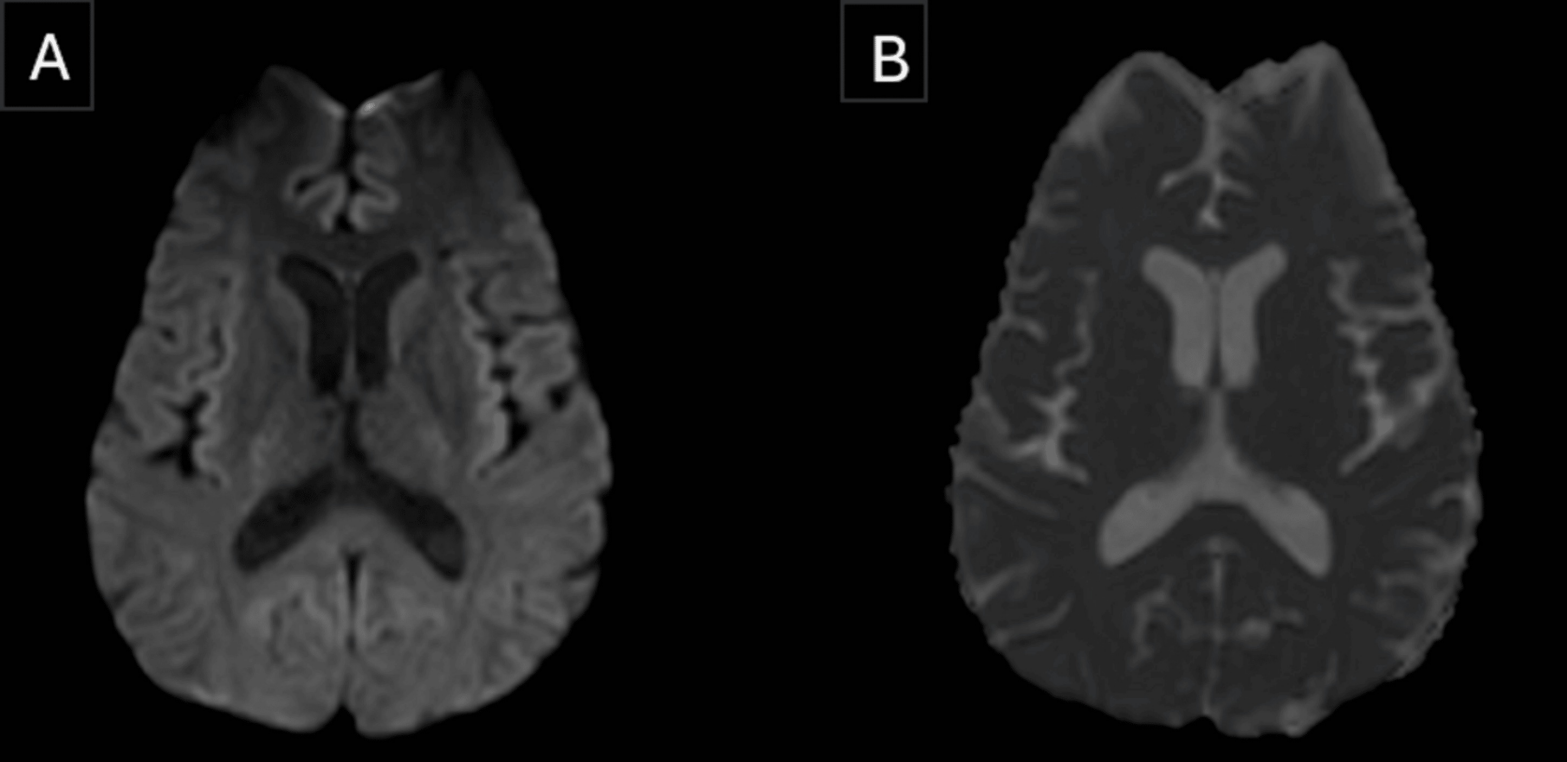
MRI Brain follow-up scan after six weeks of the initial scan. A) The diffusion-weighted imaging (DWI) sequence shows complete resolution of the prior restricted diffusion lesion at the splenium of corpus callosum seen in Figure 2A. B) The corresponding apparent diffusion coefficient (ADC) map shows complete resolution of the prior restricted diffusion lesion at the splenium of corpus callosum seen in Figure 2B.

**Figure 5:**
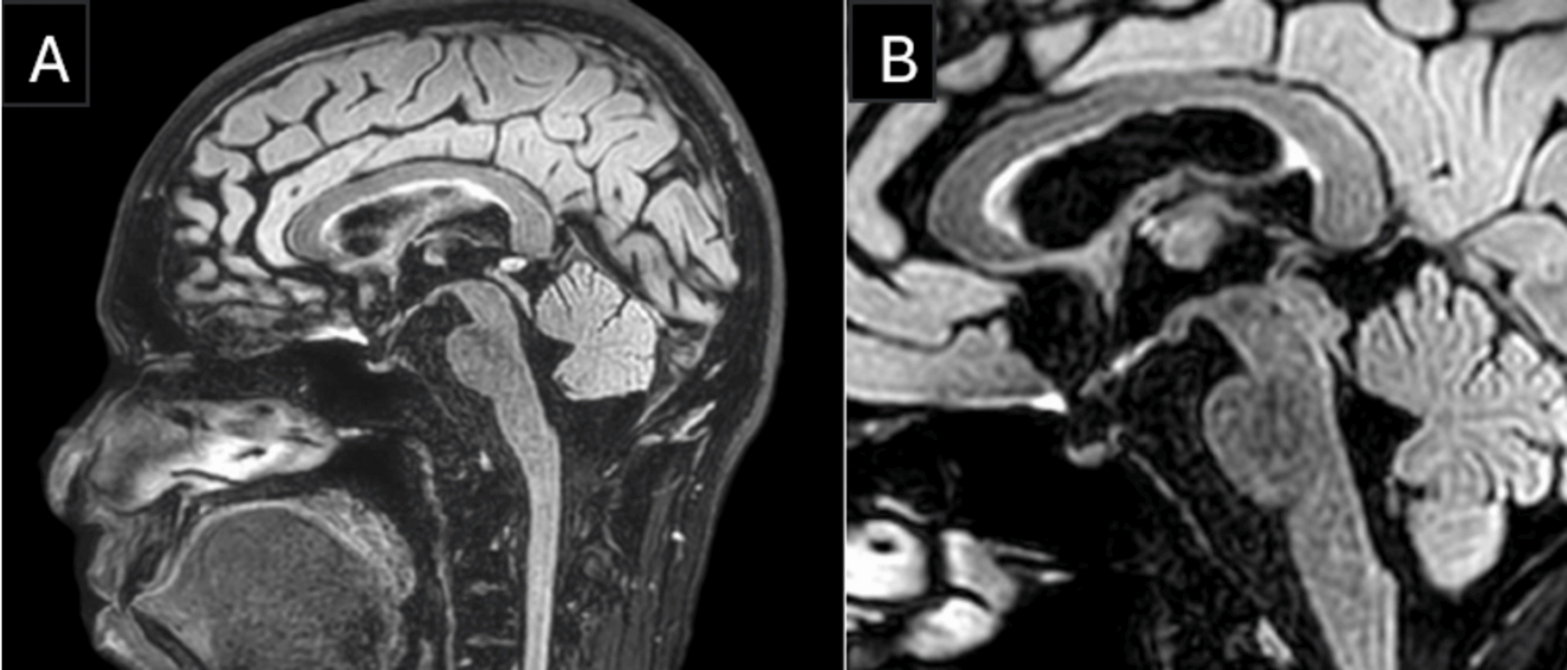
MRI Brain follow-up scan after six weeks of the initial scan. A) The Fluid-Attenuated Inversion Recovery (FLAIR) sequence (sagittal view) shows complete resolution of the prior hyperintense lesions involving the splenium of the corpus callosum seen in Figure 3A. B) The FLAIR sequence sagittal view (zoomed image) shows complete resolution of the prior hyperintense lesions involving the splenium of the corpus callosum seen in Figure 3A.

In view of the significant history of alcohol consumption and involvement of the central fibers of the splenium of the corpus callosum, a diagnosis of Marchiafava-Bignami disease was made. He was treated with intravenous thiamine (600 mg/day), benzodiazepines, other vitamin B supplements, and supportive care. Through the second day of the hospital admission, his sensorium started to improve gradually with better orientation to time, place, and person. Over a course of two weeks, he was able to walk without support. The patient was discharged with an outpatient neurology follow-up plan. Six weeks later, the outpatient consultation revealed complete remission of his symptoms with no deficits in the neurological examination. A repeat MRI scan of the brain, which was performed on the very same day, showed complete resolution of the lesions in comparison to his previous MRI brain scan (Figures 4, 5).

## Discussion

MBD is characterized by demyelination of the corpus callosum with varied clinical presentations. It was first described in 1903 by two Italian pathologists, Marchiafava and Bignami, during the autopsy of three patients who presented with status epilepticus and coma. They found that these patients had severe necrosis in the middle two-thirds of the corpus callosum. All of them had a history of chronic alcohol consumption [4]. Pathologically, MBD is characterized by symmetric demyelination and necrosis of the central part of the corpus callosum, with relative sparing of the dorsal and ventral layers [5]. Other structures that may be rarely affected include the optic chiasm and its tracts, the putamen, the anterior commissure, the cerebellar peduncles, the cortical grey matter, and the U fibers [6]. Marchiafava-Bignami disease can present in various clinical forms [7]. As there are no characteristic symptoms, it is helpful to know the clinical presentations that have been observed in various patients. Impaired consciousness, psychotic symptoms, depression, apathy, seizures, hemiparesis, ataxia, and apraxia are clinical clues to MBD in ‘at-risk’ patients [8]. The clinical course of this disease can be acute, subacute, or chronic, potentially enough to cause death within several weeks or months.

Relevant radiological findings

MBD has symmetrical and edematous patches on MRI of the brain that are confined to the corpus callosum [9]. The corpus callosum appears hyperintense on T2-weighted images, hypointense on T1-weighted images, and proton density-weighted images in the acute phase. A classical radiological sign in acute MBD is the “sandwich sign” (Figure 3A), defined by a symmetrical lesion encompassing the central region of the body of the corpus callosum while sparing the ventral and dorsal layers [10]. 

In the subacute phase, cystic lesions and small foci of hypointensity may develop on T2-weighted images, most likely due to hemosiderin deposition. In the chronic phase, signal changes are not evident and are associated with residual atrophy of the affected structures [5].

Clinical and neuroradiological classification of MBD describe two subtypes

Type A

The patients who are classified as type A would have an acute to subacute presentation with impaired consciousness, pyramidal tract signs, limb hypertonia, or seizures. The brain MRI image would show a hyperintense lesion of the corpus callosum on the T2-weighted sequences. This variant has a poor prognosis with significant morbidity and mortality rates.

Type B

In comparison with type A, this is a milder form with normal or mildly impaired consciousness, dysarthria, gait disturbances, and features of interhemispheric disconnection. In this variant, the brain MRI would show hyperintense lesions on the T2-weighted images that partially involve the corpus callosum, for example, the splenium, as in this case. The MRI lesions might regress, consequently having a favorable prognosis [11].

Differential diagnoses to consider

Acute MBD needs to be distinguished from Wernicke encephalopathy, another alcohol-related disease that has overlapping clinical findings with MBD, including ataxia and confusion. The chronic form of MBD, which could present with dementia, needs to be differentiated from Alzheimer's disease [12,13].

Some other possible diagnoses for splenial hyperintensity on the T2/FLAIR sequences are: epilepsy, drug withdrawal from antiepileptic drugs, acute disseminated encephalomyelitis, cerebral infarction, viral (especially influenza and HIV) and bacterial CNS infections, hypoglycemia, and multiple sclerosis [12].

We have excluded all the above conditions based on clinical, radiological, and laboratory data analysis.

In this case, the diagnosis of MBD type B was based on the clinical features of impaired consciousness and ataxia, along with radiological features of restricted diffusion in the DWI sequence and hyperintensity in the T2/FLAIR sequences involving the center of the splenium of the corpus callosum with dorsal-ventral sparing. Our patient had a complete recovery, both clinically and radiologically. We would like to add that, given the complete radiological resolution, the findings may be consistent with transient intra-myelinic edema, though demyelination cannot be definitively excluded without advanced imaging or histopathological confirmation.

## Conclusions

Marchiafava-Bignami disease is a rare complication of chronic alcohol consumption associated with varying clinical presentations. It was often misdiagnosed and/or undertreated in the past. However, the recent advancement in neuroimaging has facilitated early diagnosis and treatment of the condition. Clinicians should be aware that a diagnosis of Marchiafava-Bignami disease type B should be on the top of the differential list when evaluating patients who present with altered sensorium and other concomitant neurological symptoms alongside a history of significant alcohol use and a partial corpus callosal lesion in the MRI brain. Prompt recognition and management are crucial, as timely intervention can lead to improved outcomes and prevent further neurological deterioration. As in this patient, an early diagnosis guided by neuroimaging and prompt treatment involving alcohol abstinence, alcohol withdrawal therapy, administration of thiamine, and other vitamin B supplements could potentially help with complete clinical recovery, especially in MBD type B.
